# Are we Only Doing Good? Long-term Psychosocial Effects of Fertility Preservation (or Lack Thereof) on Survivors of Cancer During Adolescence and Young Adulthood

**DOI:** 10.1097/PPO.0000000000000774

**Published:** 2025-08-11

**Authors:** Vicky Lehmann, Niels van Poecke, Leah Waterman, Christianne A.R. Lok, Catharina C.M. Beerendonk, Ellen M.A. Smets

**Affiliations:** * Department of Medical Psychology; † Cancer Center Amsterdam (CCA); ‡ Department of Medical Oncology, Amsterdam University Medical Centers; § Department of Gynecologic Oncology, Center Gynaecologic Oncology Amsterdam, Antoni van Leeuwenhoek, Amsterdam; ∥ Department of Obstetrics and Gynecology, Radboud University Medical Center, Nijmegen, The Netherlands

**Keywords:** adolescents and young adults (AYA), oncology, fertility preservation, oncofertility, psychosocial effects, survivorship, reproductive goals, assisted reproductive technologies (ART)

## Abstract

**Purpose/Background:**

Patients diagnosed with cancer at reproductive age can be offered fertility preservation, which includes options of freezing sperm (male patients), oocytes, embryos, or ovarian tissue (female patients). This is intended to provide survivors with a chance to have biological children later in life (e.g., through utilizing assisted reproductive technologies, ART). However, psychosocial effects of no or completed fertility preservation remain largely unknown.

**Methods:**

A total of 48 survivors completed semi-structured interviews (M_age_ = 34 y). They had been diagnosed with cancer during adolescence and young adulthood (AYA; between age 12 and 39 y), were <1 to 18 years (M = 5 y) from diagnosis, and had completed active cancer treatment. Survivors were asked about perceived consequences of having or not having completed fertility preservation. Answers were qualitatively analyzed with template analysis.

**Results:**

Almost half of the survivors had completed fertility preservation at diagnosis. During interviews, all survivors described an emotional impact of no or completed fertility preservation, which caused positive or negative feelings, or was described as minor/absent. These feelings can change over time, as they were determined by past, present, or possibly future events. Such events clustered into a disruption in family building, followed by a phase of exploration of reproductive health posttreatment. This phase included much uncertainty, which triggered the exploration of survivors’ fertility status, reproduction/pregnancies, and options of ART. Hope for natural conception prevailed irrespective of completed fertility preservation and was still abstract for various survivors. Utilization of ART was scarce and physically and emotionally burdensome. Alternatives to biological parenthood were deemed unfeasible. Uncertainty and phases of exploration, together with learning more about their fertility status (e.g., uncovering infertility/having problems conceiving, unexpected pregnancies) changed survivors’ outlook on life and affected their romantic relationships, partner communication, and dating profoundly.

**Discussion:**

Uncertainty about fertility and reproductive options is universal, irrespective of whether survivors had completed fertility preservation or not. If completed, fertility preservation can provide survivors with positive feelings (e.g., hope/reassurance), but uncertainties and worries surrounding reproduction/ART can add substantial burden throughout survivorship. Survivors’ perception of no/completed fertility preservation can change over time and largely depends on whether ART is needed and its outcome. Thus, fertility preservation cannot always buffer negative effects, and if survivors remain without (additional) children unintentionally, emotional burden and grief can be significant. Health care providers should address any concerns of AYA patients/survivors and counsel them realistically about family building options; and refer patients and survivors to mental health specialists, if needed.

Cancer treatment during adolescence and young adulthood (AYA; defined as age 15-39 y),^1^ can affect fertility. Gonadotoxic cancer therapies (e.g., alkylating chemotherapy, radiation, or surgery of reproductive organs) put 20% to 60% of patients at risk for infertility, depending on type of cancer and treatments and reaching even >90% in patients who received stem cell transplants.^2–7^ Moreover, the timing of a cancer diagnosis itself can hinder patients’ family planning, even if fertility is unaffected by treatment (e.g., due to delaying dating experiences).^8^ Clinical guidelines,^1,9–12^ the Oncofertility Consortium,^13^ and patient advocate organizations^14–16^ recommend offering fertility preservation (FP, i.e., freezing sperm/oocytes/embryos/gonadal tissue) to young cancer patients, if possible. This provides a possibility for biological parenthood later in life using assisted reproductive technologies (ART). Yet, it remains unknown if FP can buffer negative psychosocial effects of (possible) infertility in survivors.

Most survivors appreciate fertility-related information at diagnosis.^17–21^ Feeling informed can reduce fertility-related concerns later in life.^18,22,23^ However, not every patient pursues FP due to various reasons (e.g., time constraints, tumor location, personal choice)^24–26^ and some survivors can experience regret in hindsight.^18,20,27–32^ At the same time, little is known about psychosocial effects among survivors who completed FP, except that they regard FP as a safeguard.^33,34^ Another study comparing survivors with versus without completed FP showed that both groups experience similar levels of fertility-related distress,^35^ which prompts the question whether completing FP is always advantageous.

Many cancer survivors want to become parents, but they are less likely to conceive and have children than controls.^36,37^ If survivors have limited or no options to have biological children, it can have profound effects on their dating behaviors and romantic relationships: qualitative studies indicated that survivors worried about not finding a partner or experienced break-ups due to infertility, although other survivors found joy in a life without children.^17,38–41^ Besides fertility problems, concerns about survivors’ own or a potential offspring’s health may play a role,^22,42,43^ but whether such concerns actually stop survivors from trying to have children remains unclear. Few survivors utilize their preserved materials through ART later in life (<13%),^44–49^ but rates may increase over time as survivors grow older.^50^ Underlying reasons for low ART utilization are understudied, but include natural conception (making ART redundant), young age/maturity, or death.^44,49,51^

Research about the perceived consequences of having versus not having completed FP at diagnosis and whether FP can buffer negative psychosocial effects of (potential) infertility in survivorship is missing. Therefore, this qualitative study examined perceived consequences on family planning and romantic relationships in survivors of AYA cancer.

## METHODS

### Study Design and Procedures

Data are part of the FROSA study,^52^ which utilized online convenience sampling targeting Dutch-speaking survivors of AYA cancer, who had completed active treatment. FROSA consisted of an online survey (completed by 190 participants) and an optional interview by phone or video call, which was completed by 48 survivors and included in the current analyses.

Participants provided oral consent for their interview participation during scheduling and at the start of the interview. Almost all participants also consented to their interview being recorded, except in 2 cases where the interviewer took extensive notes. Interviews were done by one trained interviewer (L.W., research assistant/psychologist), lasted between 20 and 60 minutes, and were semistructured (including broad questions to have in-depth conversations about lived experiences). Analyses focused on interview questions about what it meant for survivors’ lives that they have or have not completed FP (Appendix-1, Supplemental Digital Content 1, http://links.lww.com/PPO/A44). We also noted participants’ sex, relationship status (at diagnosis and interview), parental status (at diagnosis and interview), age at and type of diagnosis. Our medical ethical committee approved this study (W21_282#21.309), all procedures were in line with the Declaration of Helsinki, and analyses/reported results adhered to the COREQ guidelines.^53^

### Analyses

Interviews were transcribed verbatim and analyzed in the software program MaxQDA by means of Template Analysis, which is a form of thematic analysis that starts by building a (hierarchical) template based on coders familiarizing themselves with the data.^54^ This was done by the project leader (V.L.) and interviewer (L.W.), who identified a temporal nature of perceived consequences (e.g., survivors described how they were affected in the past, but not present). This led to developing a template for coding perceived consequences of no or completed FP divided into past, present, and possible future perceived consequences. The salient content of these 3 parts was coded entirely open and inductively (i.e., bottom-up) based on participants utterances. Possible labels and content-based codes were added to the template as needed throughout coding.^54^

Two experienced coders [V.L. (psychologist, female), N.v.P. (sociologist, male)] independently coded 5 interviews to test the initial template and added content-based codes. These were discussed before coding another 8 interviews and discussing the content. Both coders identified the same content as relevant. Therefore, one coder (V.L.) coded all remaining interviews, which were discussed (V.L., L.W., and N.v.P.) to minimize subjectivity. In further analyzing our data, all identified codes were further categorized and regrouped as needed, and overarching themes were identified and discussed among all authors. Initial results were presented to 11 interviewees (55% females) during 2 in-person participant validation meetings (i.e., a technique to double-check results for accuracy with original participants).^55^ Based on these meetings, no themes were changed, but nuances to the descriptions were added.

Qualitative analyses typically reach saturation after analyzing data of 15 to 20 participants,^56^ but we analyzed all interviews as each survivor’s timeline and experiences had unique aspects. Consequently, we continued to add codes across all interviews, but codes that were identified only once were deemed incidental and not reported.

## RESULTS

The 48 survivors were 34.2 years old and 4.8 years post diagnosis. Most common types of diagnoses included breast cancer (17, 35.4%) and leukemia/lymphoma (11, 22.9%). Most survivors were females (36, 75.0%) and partnered (39; 81.3%) at the time of interviews. A total of 22 (45.8%) participants had completed FP at diagnosis, which included 12 women (33.3% of all female survivors) and 10 men (83.3% of all male survivors; Table 1).

**TABLE 1:** Descriptive Statistics of All Participants (N = 48)

	All Survivors	Female	Male
	N = 48	n = 36	n = 12
Age at study, M (SD), range	34.2 (5.2), 20-49	34.4 (4.8), 26-49	33.5 (6.4), 20-44
Age at first diagnosis (y); n (%)			
≤12	1 (2.1)	1 (2.8)	—
13-17	2 (4.2)	—	2 (16.7)
18-24	9 (18.8)	5 (13.9)	4 (33.3)
25-29	13 (27.1)	9 (25.0)	4 (33.3)
30-34	16 (33.3)	15 (41.7)	1 (8.3)
35-39	7 (14.6)	6 (16.7)	1 (8.3)
Age at most recent diagnosis^*^, M (SD), range	28.5 (5.2), 18-38	29.5 (4.7), 19-38	25.7 (5.9), 18-38
Years since most recent diagnosis, M (SD), range; n (%)	4.8 (4.6), 0-18	4.7 (4.5), 0-18	4.8 (5.0), 1-17
<2	12 (25.0)	11 (30.6)	1 (8.3)
2-3	15 (31.3)	7 (19.4)	8 (66.7)
4-5	6 (12.5)	6 (16.7)	—
6+	6 (12.5)	5 (13.9)	1 (8.3)
10+	9 (18.8)	7 (19.4)	2 (16.7)
Relationship status; n (%)			
Single	7 (14.6)	5 (13.9)	2 (16.7)
Single at dx and now	2 (4.2)	2 (5.6)	—
Partnered at dx, single now	5 (10.4)	3 (8.3)	2 (16.7)
Partnered	41 (85.4)	31 (86.1)	10 (83.3)
Still same partner	26 (54.2)	22 (61.1)	4 (33.3)
Partnered at dx, new partner now	9 (18.8)	5 (13.9)	4 (33.3)
Single at dx, partnered now	6 (12.5)	4 (11.1)	2 (16.7)
Type of diagnosis; n (%)			
Breast cancer	—	17 (47.2)	—
Lymphoma/leukemia	—	7 (19.4)	4 (33.3)
Gynecological cancers	—	6 (16.7)	—
Testicular cancer	—	—	5 (41.7)
Sarcoma	—	3 (8.3)	—
Other	—	3 (8.3)	3 (25.0)
Fertility preservation (FP) at diagnosis; n (%)			
No attempt	23 (47.9)	21 (58.3)	2 (16.7)
Attempted FP	—	2 (5.6)	—
Completed FP^†^	22 (45.8)	12 (33.3)	10 (83.3)
Fertility-sparring through hormones	—	1 (2.8)	—
Parental status; n (%)			
No biological children	26 (54.2)^‡^	18 (50.0)	8 (66.7)
Ongoing pregnancy (no prior children)	1 (2.1)	1 (2.8)	—
Had biological children:	21 (43.7)^§^	17 (47.2)	4 (33.3)
Before dx	12/21 (57.1)	10/17 (58.8)	2/4 (50.0)
After dx	8/21 (38.1)	6/17 (35.3)	2/4 (50.0)
Before and after dx	1/21 (4.8)	1/17 (5.9)	—

^*^ n = 11 had a relapse/second malignancy.

^†^ Included freezing n = 7 oocyte, n = 4 embryo, n = 1 ovarian tissue (women), and n = 10 fresh semen (men).

^‡^ n = 1 had stepchildren.

^§^ n = 2 had biological and stepchildren.

dx indicates diagnosis.

Experiences of no or completed FP clustered into 5 themes and several subthemes (see Table 2 and Appendix-2, Supplemental Digital Content 1, http://links.lww.com/PPO/A44). At diagnosis, a (1) disruption in family building was salient. Once treatment was completed, (2) reproductive health became a concern, which included (2.1) uncertainty about fertility, and exploring one’s (2.2) fertility status, (2.3) reproduction, and (2.4) ART. All aspects could change survivors’ (3) outlook on life and affect their (4) romantic relationships/dating. Perceived consequences were dependent on previous, ongoing, or possible future experiences and resulted in a currently perceived (5) emotional impact at the time of interviews. This impact was either (5.1) positive, (5.2) negative, or (5.3) minimal/absent and all themes are described below including brief quotes in quotation marks (see longer quotes in Appendix-3, Supplemental Digital Content 1, http://links.lww.com/PPO/A44).

**TABLE 2:** Overview of Identified Themes, Subthemes and Examples; Together With Contributing (Background) Factors for Each (Sub)Theme

		Contributing Factors
Past, present, and possible future consequences		
1.	Disruption of family planning (trying to conceive, being pregnant, being postpartum, miscarriages)	Older age, relationship status: partnered
2.	Reproductive health following treatment	
	2.1 Uncertainty causing:	Sex: female, strong desire*
	- Preoccupation, concerns, sadness vs hope	
	- Doubts reproductive goals	
	→ 2.2 Exploring fertility	Symptoms of possible infertility, new partners
	- Fertility testing - Contemplate alternatives to biological parenthood, stepparent	
	→ 2.3 Exploring reproduction and pregnancies - Worries (age, own health, offspring health, safe pregnancies, breastfeeding)	Desire*, age, health status, sex: female type of cancer (breast), infertility
	- Resolved: spontaneous pregnancies - Unintended pregnancies, contraceptives, hormone therapy	
	→ 2.4 Exploring ART	Possible/confirmed infertility time, health status, sex: female†, outcome, ART
	- Welcome, but abstract option - Challenging and strenuous process	
3.	Different future and outlook on life (adjust life goals)	Time, infertility, desire*
4.	Relationship impact	
	4.1 Positive and negative effects	Ongoing vs former relationships
	(being stronger/closer together vs being on a different page)	
	4.2 Partner communication	Relationship status: partnered, social environment, desire*
	Neutral vs positive about goals, reproductive options/ART	
	Professional help	
	4.3 Dating	Relationship status: single
	- Concerns, disclosure	
Present resulting consequences		
5.	Emotional impact	
	5.1 Positive feelings	Completed FP, strong desire*, successful ART conscious rejection FP
	Hope, happiness, peace of mind → Less (time) pressure, reassurance, backup plan,	
	5.2 Negative feelings	No/failed FP, limited frozen material, failed ART, infertility, social environment
	Sadness, grief, disappointment, loss, pain	
	5.3 Minimal or absent emotional impact, determined by:	Parental status, time since treatment, no/low desire*, younger age, relationship status: single
	5.3.1 Completed family building (any consequences, if present, resolved in the past)	
	5.3.2. No priority: (a) No desire to have any children (b) Reproductive goals open possibly for future	

* Refers to desire to have children.

† ART was regarded as more strenuous process for female survivors and female partners of male survivors.

### Disruption of Family Planning

The timing of diagnosis can disrupt family building by either delaying plans (causing feelings of “sadness” and feeling “put on hold”) or by coinciding with family building. The latter included actively trying to conceive, being pregnant, being postpartum, or having had miscarriages around the time of diagnosis. Such circumstances made the cancer diagnosis itself, consideration of reproductive goals, and decisions around FP “surrealistic” and “difficult to grasp.” Moreover, being told at diagnosis to certainly become infertile, resulted in having to abandon reproductive goals and intense “anguish,” “anxiety,” and “heartache.”

### Reproductive Health Following Treatment

#### Uncertainty

Once treatment was completed, phases of uncertainty became salient including feeling uncertain about (in)fertility and safe future pregnancies. Uncertainty led to concerns and preoccupation with fertility, feelings of “sadness,” “frustration”, and was sometimes experienced as “worse than cancer.” Female survivors were sometimes faced with uncertainty up to 10 years due to antihormonal therapies.

Although facing uncertainty, survivors remained hopeful about being fertile, while the possible prospect of more difficult ways of family building (e.g., ART) also increased doubts about reproductive goals. It became a more complex choice. Sometimes, participants experienced no uncertainty when they trusted their oncologists’ prediction (e.g., to remain fertile).

#### Exploring Fertility

An uncertain fertility status together with symptoms of possible infertility (e.g., amenorrhea) or new romantic relationships, led to exploring or testing fertility to regain control/minimize ambiguity. Reactions to fertility assessments varied from relief over intact fertility, continued uncertainty due to unclear results, suspicion due to positive results without siring a pregnancy, or little impact of confirmed infertility due to having cryopreserved materials as “backup plan“. In contrast, confirmed or certain infertility also caused feelings of “loss” and “pain.”

When exploring their fertility status, survivors felt sometimes frustrated that the (in)ability to have children was overemphasized by providers. Instead, survivors also wanted a focus on related aspects of hormonal imbalances and implications on daily life (e.g., daily testosterone replacements, hot flushes/menopausal symptoms, bone density).

Uncertainty about fertility also led to the contemplating alternatives to biological parenthood (i.e., adoption/fostering, donation/surrogacy), but was often deemed as too complex and impossible/unrealistic (legally, financially, and emotionally). No participant had pursued such alternatives, and some were happy about another alternative: becoming a stepparent.

#### Exploring Reproduction and Pregnancies

When considering to possibly have children, various reservations, worries, and concerns were described. This included worries about increasing age and timing (e.g., missing their reproductive window), which increased the pressure to have children earlier. Moreover, worries about the health and genetic risk of cancer for a potential child, and worries about survivors’ own health, safe pregnancies and breastfeeding (in female survivors) were common. Such health-related worries also led to doubting reproductive goals.

Spontaneous natural conception resolved uncertainties or worries, causing relief and letting go of any possible concerns or pressure once children were born. Survivors regarded confirmed infertility as making contraceptive use superfluous, but unintended pregnancies also occurred if survivors refrained from using contraceptives after being told their fertility was compromised.

Some female (breast cancer) survivors paused adjuvant hormone therapy to try and become pregnant. Such pause caused worries about cancer recurrence, and stress, because women felt pressure to become pregnant quickly, to restart hormone therapy after pregnancy.

#### Exploring Assisted Reproductive Technologies

When facing uncertain fertility or confirmed infertility, ART options were explored. If hypothetically considering future ART, it was a welcome alternative to natural conception, but remained abstract. If ART represented the only remaining option to reproduce, it was regarded as very positive. Yet, in both cases, ART represented another source of uncertainty and caused worries due to the complexity and limited success rates of ART procedures. Sometimes, ART was perceived as an unrealistic option (e.g., due to hormone injections and assumed risk for cancer relapse).

Survivors who previously or currently pursued ART described it predominantly as “emotionally challenging”, “hard,” and “strenuous process,” especially if it repeatedly failed. A related concern included worries about limited amounts of frozen materials, because it extrapolated limited chances to have biological children, made each failed ART cycle a greater disappointment, and increased worries about family planning. If ART remained unsuccessful, survivors experienced extensive emotional burden, disappointment, and relationship dissolutions, which also resulted in concluding never to do ART again. ART procedures also had negative effects on couples’ sex lives, which were absent or described as “less romantic” or “planned.” If ART was successful, it resulted in being “thankful” for FP in hindsight.

Thus, it appeared that lived experiences of FP and ART represented 2 opposites depending on the outcome of ART. The greater physical and mental burden of ART for female survivors and (future) female partners of male survivors was emphasized (Appendix-3, Supplemental Digital Content 1, http://links.lww.com/PPO/A44).

### Outlook on Life

Adjusting life goals was common, as (potential) fertility problems presented survivors with a differently envisioned future. It resulted in having no or fewer children or deciding to have children earlier. Health care providers sometimes advised to have children “sooner rather than later,” which made individual situations more complex if survivors could not act accordingly (e.g., young age, other priorities). Those who unintentionally remained without any/additional children, experienced this as a painful process of acceptance.

For other survivors, the future and possible effects remained unclear, especially if reproductive goals were undecided. If they wanted to have children in the distant future, survivors postponed exploring their reproductive options (e.g., once hormone treatments are completed, once they established committed relationships, once they are older/feel more mature).

### Relationship Impact

#### Positive and Negative Effects

Effects on romantic relationships varied. Positive effects were described when survivors were still together with the same partner, which included descriptions of “stronger” or “closer together” while emphasizing the importance of “moving forward together” and communicating (see below). In contrast, negative experiences were described after break-ups or when the future of a relationship was uncertain. Negative effects on romantic relationships included “being on a different page” or “growing apart” when discussing reproductive goals, ART, or coping with (potential) infertility. The latter was experienced as survivors’ “own pain.”

#### Partner Communication

If partnered, survivors reported regular partner communication about family planning, often prompted by pregnancies in their social environment. Such conversations sometimes led to realizing that partners had different or changed reproductive goals over time4. Descriptions of communication about family planning in the past varied in tone: it was either neutrally mentioned that conversations occurred or they were positive. In the latter situation, partners were supportive and involved. Although such conversations can be hard, survivors expressed that they were necessary. This was sometimes supported by seeking professional help.

Possible ART was also discussed within couples and partners were open to such options. Yet, survivors also indicated feelings of “guilt” or “regret” about more complex ways of getting a child or not being able to “provide a child” to a partner. Survivors also remained insecure even if partners reassured them that they prioritized a life with them over having children.

#### Dating

If unpartnered, feelings of inferiority or concerns about (possible) infertility or ART occurred when dating. Worries included that dating partners would be uninterested and discouraged to continue dating if finding out about cancer and (potential) infertility. Consequently, survivors wondered when and how to disclose their history. Different strategies included being upfront or disclosing information in several steps to also being able to determine for themselves if survivors felt comfortable with their dates. Overall, open and honest communication was emphasized, so that dating partners can also make informed decisions about further committing to a possible relationship. Disclosure sometimes resulted in not further pursuing a date, from both sides.

### Current Emotional Impact

The above past, current and possibly future experiences led survivors to describing an emotional impact of having or not having completed FP on their current lives in 3 different ways.

#### Positive Feelings

Having completed FP led to positive feelings, including “hope,” “happiness,” and “peace of mind.” It presented survivors with “more time” or “less pressure” to have children. Such positive views particularly prevailed if a desire to have children in the future was strong. Accordingly, having completed FP was labeled as “reassurance/insurance,” “backup plan,” or “choice” for the future. However, hope for natural conception and not needing ART prevailed. These positive feelings were also salient in survivors who had children through ART, which represented their only option to have biological children and for which they were “thankful.” If survivors felt they made an informed decision about not completing FP, they also felt positively about it, reporting acceptance.

Although mainly positive, some past events led to changing attitudes toward FP. For example, if ART became a necessity, positive feelings about FP changed to feeling emotional upset given that ART comes with its own challanges (as described above; see also Appendix-3, Supplemental Digital Content 1, http://links.lww.com/PPO/A44).

#### Negative Feelings

Predominantly negative feelings occurred if FP was impossible, had failed, if preserved materials were limited, or if complications occurred (e.g., painful ovarian overstimulation/extensive bloating in women). Negative feelings included “sadness,” “grief,” “disappointment,” and “loss,” which referred to fertility itself or a choice about parenthood being taken away. These survivors felt ill-informed and unprepared for possible side effects or that FP could even fail. They felt they lacked “a realistic picture of what to expect” of FP procedures or their future fertility. Such negative feelings were particularly present if the desire to have (additional) children in the future was strong.

Over time, negative feelings were reinforced every time when pregnancies were announced in survivors’ social environment. Unsolicited questions about having or wanting to have children were experienced as painful. Survivors also became more critical or doubtful about their reproductive goals. They questioned whether they would be able to parent a child mentally and physically.

#### Minimal/Absent Emotional Impact

If (5.3.1) family building was completed in the past (before and/or following cancer treatment), survivors perceived no or minimal impact of having or not having completed FP. If they had conceived after treatment, pregnancies had resolved any possible worries about fertility (i.e., spontaneous pregnancies reassured their intact fertility).

If (5.3.2) fertility/family planning had no priority, survivors perceived no or minimal consequences at the time of interview. This was either due to (1) no desire to having children and accepting/choosing a life without children, or (2) reproductive goals still being open/something for the future. The latter was the case if survivors were still young, single, and/or had more recently completed cancer treatment. Family building was still too abstract.

While a fulfilling life without children was certainly a feasible option, it was sometimes accompanied by a difficult process of emotional acceptance. At the same time, not having completed FP and being infertile following treatment also made life easier as it took away any uncertainty or worry.

## DISCUSSION

Fertility preservation (FP) is offered with good intentions to cancer patients, but long-term psychosocial effects of no or completed FP are barely studied. Instead, fertility-related distress has been established in survivors regardless of whether they completed FP or not,^35^ and our study provided more in-depth insights: survivors experience a variety of emotional effects which can change over time. Such change occurs in conjunction with survivors’ exploration of their fertility status and subsequent reproductive options; and the answer to the question “Can completed FP buffer negative effects of (possible) infertility later in life?” is multifaceted.

First, uncertainty in itself can be an unpleasant emotional state, especially if individuals have a low tolerance to cope with uncertainty.^57^ We found that uncertainty is universal among survivors and cannot entirely be buffered by having frozen materials, because (1) survivors hope for natural conception regardless of whether they completed FP, or because (2) preserved materials in itself provide additional sources of uncertainty and concern (e.g., whether preserved material is sufficient, ART is safe or successful). Notably, the hope for natural conception differs in its nuance between survivors who have completed FP (i.e., hope for natural conception, while having preserved material as back-up) and survivors without FP (i.e., hope for natural conception as only option for biological children). Such hope may change or vanish over time once survivors start to explore and learn more about their fertility status posttreatment, which further determines their reproductive options.

Second, perceiving completed FP as positive or negative depends on the outcome of ART or natural conception (making ART redundant). Thus, as long as family planning remains uncompleted (i.e., having children or deciding against it), cryopreserved materials present survivors with a double-edged sword:

- At the time of cancer diagnosis, FP offers hope and future reproductive options, but entails additional medical procedures, uncertainty, and distress, especially if FP fails or if limited material is preserved. The latter was unexpected for patients (e.g., they were inadequately informed/forgot). In contrast, knowing about FP, but being unable to complete it, causes negative feelings.
- After cancer treatment, FP offers hope and additional reproductive options, but it is a backup plan filled with uncertainty and false hope if utilizing preserved materials through ART does not work; ART itself is a burden with extensive impact on relationships. In contrast, not having completed FP may provide survivors with more time to cope and accept no or limited reproductive options.

FP can certainly provide hope and a backup plan,^33,34^ but needs to be balanced against limited ART success rates of 10% to 30% per IVF cycle.^58^ For participants who utilized ART, it represented a physically and emotionally strenuous process. If unsuccessful, it ended in relationship dissolutions, comparable to couples with unexplained fertility problems.^59,60^ If successful, survivors regarded FP as very positive in hindsight. For survivors with fertility problems who did not complete FP at diagnosis, alternatives to biological parenthood were mentioned by providers, but survivors found these options unrealistic (e.g., commercial surrogacy is illegal in the Netherlands), which often caused additional emotional burden. This is different from US-based studies, where survivors considered alternatives to biological parenthood as realistic alternatives,^61–64^ although some survivors may be oblivious to the procedures and financial means needed for these alternatives.

Third, whether, when, and how preserved materials or lack thereof affect survivors is further determined by background or situational factors (see also Table 2), which can change over time.

- Survivors’ relationship status can determine currently perceived effects. For example, single survivors experienced less emotional impact given that family planning was too abstract. Yet, some worried about not finding a partner if infertile, as also demonstrated in previous research.^39,41^ Partnered survivors sometimes experienced guilt about possibly not being able to “provide” a child to a partner, as also reported previously.^38,41^ Importantly, relationship status can change over time due to infertility or disagreement about reproductive goals. Such identified effects support earlier research of couple’s difficulties communicating about fertility^41^ versus bringing them closer together.^65^ Yet, our study also showed that preserved material itself can be perceived as backup plan for both partners and survivors, but that partners may also change their opinions over time (e.g., about ART/alternatives).
- Age together with time since treatment determined whether possible consequences of no or completed FP may still lay ahead of survivors in the future (e.g., young age),^49^ were resolved in the past (e.g., completed family building posttreatment), or were ongoing (e.g., feeling rushed to have children due to advancing age).
- Reproductive goals determined the intensity of perceived consequences. For example, survivors experienced minimal impact if they did not want to have children or wanted to have them in the distant future. In contrast, others worried intensely if having (any/more) children was an important life goal.
- Female survivors expressed worries about the safety of becoming pregnant, possibility to breast feed, and the invasiveness of (possible) ART procedures, especially after having gone through cancer. This offers a possible explanation to previous research that showed male cancer survivors being more likely to utilize ART with female partners, than female survivors.^49^ Nevertheless, male survivors in our study also expressed guilt toward possibly burdening a (future) female partner with ART. Thus, the physical burden of ART is experienced as greater in women, but worries around it are present in both male and female survivors. Moreover, the timing of trying to become pregnant for female breast cancer patients may be hindered by extended administration of adjuvant hormone therapies following active cancer treatment.
- The effects of parental status at diagnosis varied. Survivors had sometimes completed their family building before diagnosis and experienced little or no effects. In contrast, others who also already had children grieved/were still very negatively affected by not having completed FP and possibly becoming infertile, because they had a strong desire to have more children. Thus, having had children before an AYA cancer diagnosis does not prevent survivors from experiencing negative effects of not completing FP.^18^ Obviously, different combinations of the above factors can further determine survivors’ individual reactions to having or not having preserved materials and associated reproductive options. This should be considered in clinical practice.

Considering the above, it is essential to adequately inform patients about FP procedures, ART options, and alternatives to biological parenthood. This allows patients to make informed decisions,^66–68^ and to manage their expectations. Our study demonstrated that if survivors felt they made an informed decision to decline FP, they also felt positively about it. Our study and previous research^35,41–43,69–71^ demonstrated that most survivors expressed fertility-related concerns regarding their fertility potential, ART, as well as their own and a possible child’s health. Interestingly, worries about survivors' fertility potential were resolved once children were born, which confirmed survivors’ intact fertility and was combined with a relief about having a healthy baby. However, continued worries about a child’s risk to develop cancer were not expressed and how survivors cope with possible worries about their children’s health over time needs further investigation. Overall, patient-centered fertility care,^72^ focusing on uncertainty and various concerns,^73^ and including tailored and repeated consultations are needed.^33^ Thereby, discussing contraceptive use is also warranted: some survivors had unintended pregnancies after being told they were (likely) infertile. Overall, we urge providers to openly address the topic of reproductive goals: providers should not assume that every survivor wants to have children but rather emphasize that reproductive goals entail a choice. Thereby, providers should not overemphasize (in)fertility as part of reproductive health, but also counsel survivors about other implications of infertility/hormonal imbalances (e.g., daily use of testosterone replacements, hot flushes/menopausal symptoms, lower bone density).

### Limitations

Due to recruitment through online convenience sampling, we were unable to calculate response rates and likely introduced a selection bias toward survivors interested in FP/family building, while also overrepresenting female survivors. Coincidentally, we included almost equal numbers of survivors who had and had not completed FP, which was advantageous for our analyses but does not represent a realistic distribution in the AYA cancer population (i.e., FP is typically lower, especially in women). Survivors reported their currently perceived effects, but recall of past emotional states may have been susceptible to recall bias. Our interview setting over phone/video call may diminish building rapport, but participants also appreciated the privacy of their home during participation. Although adding depth and robustness to our findings, the method of participant validation in a group setting has been criticized previously (e.g., dominance of certain participants),^74^ which we minimized throughout by having small groups and continuously checking results with each participant. A sample size of 48 may be regarded as small, although it is rather large for qualitative research, which further contributed to rich findings. Finally, having examined a Dutch-speaking sample with good health insurance coverage may also contribute to the fact that few participants mentioned financial aspects regarding their family building options. This may be more prominent in other (lower-income) countries, medical settings, and cultural contexts, which requires more research.

## CONCLUSION

Our study presents the lived experiences of survivors of AYA cancer with and without completed FP at diagnosis. Future research may quantify the prevalence of all identified experiences. For now, it appears that FP has the power to offer hope, while coming at a cost of additional uncertainties and worries. Attitudes toward FP largely depend on whether ART is needed in survivorship and its outcome. Throughout survivorship, providers should discuss reproductive goals and options with survivors to provide a realistic picture of available options, while also being mindful of survivors choosing a life without children. Addressing uncertainty should be an essential part of such discussions. If needed, health care providers should refer young cancer patients and survivors to mental health specialists for support in coping with uncertainty and concerns, adjusting to a different outlook on life, grieving over missed reproductive opportunities, and effects on romantic relationships and dating.

## Supplementary Material

## References

[1] Coccia PF Pappo AS Altman J. Adolescent and young adult oncology, version 2.2014. J Natl Compr Canc Netw. 2014;12:21–32. 24453290 10.6004/jnccn.2014.0004

[2] Franasiak JM Scott RT. Demographics of cancer in the reproductive age female. In: Sabanegh ES, ed. Cancer and Fertility. 1st edn. Switzerland: Springer; 2016:11–19.

[3] Khurana KK Alukal JP. Demographics of cancer in the reproductive age male. In: Sabanegh ES, ed. Cancer and Fertility. 1st edn Switzerland: Springer; 2016:1–10.

[4] Magelssen H Brydøy M Fosså SD. The effects of cancer and cancer treatments on male reproductive function. Nat Clin Pract Urol. 2006;3:312–322. 16763643 10.1038/ncpuro0508

[5] Van Dorp W Haupt R Anderson RA. Reproductive function and outcomes in female survivors of childhood, adolescent, and young adult cancer: a review. J Clin Oncol. 2018;36:2169. 29874135 10.1200/JCO.2017.76.3441 PMC7098836

[6] Meacham LR Burns K Orwig KE. Standardizing risk assessment for treatment-related gonadal insufficiency and infertility in childhood adolescent and young adult cancer: the pediatric initiative network risk stratification system. J Adolesc Young Adul. 2020;9:662–666. 10.1089/jayao.2020.0012 32456570

[7] Van den Berg M van Dijk M Byrne J. Treatment-related fertility impairment in long-term female childhood, adolescent and young adult cancer survivors: investigating dose-effect relationships in a European case-control study (PanCareLIFE). Hum Reprod. 2021;36:1561–1573. 33744927 10.1093/humrep/deab035

[8] Ussher JM Perz J The Australian Cancer and Fertility Study Team A. Threat of biographical disruption: the gendered construction and experience of infertility following cancer for women and men. BMC Cancer. 2018;18:250. 29506492 10.1186/s12885-018-4172-5 PMC5836444

[9] Ethics Committee of the American Society for Reproductive Medicine (ASRM). Fertility preservation and reproduction in patients facing gonadotoxic therapies: an Ethics Committee opinion. Fertil Steril. 2018;110:380–386. 30098684 10.1016/j.fertnstert.2018.05.034

[10] The Practice Committee of the American Society for Reproductive Medicine Pfeifer S Goldberg J McClure R. Mature oocyte cryopreservation: a guideline. Fertil Steril. 2013;99:37–43. 23083924 10.1016/j.fertnstert.2012.09.028

[11] Oktay K Harvey BE Partridge AH. Fertility preservation in patients with cancer: ASCO clinical practice guideline update. J Clin Oncol. 2018;36:1994–2001. 29620997 10.1200/JCO.2018.78.1914

[12] Anderson RA Amant F Braat D. ESHRE guideline: female fertility preservation. Hum Reprod Open. 2020;2020:hoaa052. 33225079 10.1093/hropen/hoaa052 PMC7666361

[13] Woodruff TK Ataman-Millhouse L Acharya KS. A View from the past into our collective future: the oncofertility consortium vision statement. J Assist Reprod Genet. 2021;38:3–15. 33405006 10.1007/s10815-020-01983-4 PMC7786868

[14] Buchacz A Farrington T Gudger B. White Paper on the Needs of Young People Living With Cancer: 5 Calls to Action Brussels. Belgium: Youth Cancer Europe; 2018. Accessed April 2025. https://www.youthcancereurope.org/wp-content/uploads/2018/10/YouthCancerEurope_Brussels_2018_WhitePaper-sm.pdf

[15] AFP. Alliance of fertility preservation. Accessed April 2025. https://www.allianceforfertilitypreservation.org/about-the-alliance/

[16] Omesi L Narayan A Reinecke J. Financial assistance for fertility preservation among adolescent and young adult cancer patients: a utilization review of the Sharing Hope/LIVESTRONG fertility financial assistance program. J Adolesc Young Adul. 2019;8:554–559. 10.1089/jayao.2018.0151 31070493

[17] Crawshaw MA Sloper P. ‘Swimming against the tide’ - the influence of fertility matters on the transition to adulthood or survivorship following adolescent cancer. Eur J Cancer Care (Engl). 2010;19:610–620. 20088919 10.1111/j.1365-2354.2009.01118.x

[18] Deshpande NA Braun IM Meyer FL. Impact of fertility preservation counseling and treatment on psychological outcomes among women with cancer: a systematic review. Cancer. 2015;121:3938–3947. 26264701 10.1002/cncr.29637

[19] Garvelink MM ter Kuile MM Bakker RM. Women’s experiences with information provision and deciding about fertility preservation in the Netherlands: ‘satisfaction in general, but unmet needs’. Health Expect. 2015;18:956–968. 23647741 10.1111/hex.12068 PMC5060855

[20] Chan JL Letourneau J Salem W. Regret around fertility choices is decreased with pre-treatment counseling in gynecologic cancer patients. J Cancer Surviv. 2017;11:58–63. 27480882 10.1007/s11764-016-0563-2 PMC5269477

[21] Stein DM Victorson DE Choy JT. Fertility preservation preferences and perspectives among adult male survivors of pediatric cancer and their parents. J Adolesc Young Adul. 2014;3:75–82. 10.1089/jayao.2014.0007 PMC4048980 24940531

[22] Young K Shliakhtsitsava K Natarajan L. Fertility counseling before cancer treatment and subsequent reproductive concerns among female adolescent and young adult cancer survivors. Cancer. 2019;125:980–989. 30489638 10.1002/cncr.31862 PMC6402962

[23] Zeidman A Davis AM Ford JS. Perceptions of infertility and reproductive concerns in adolescent and young adult female cancer survivors. J Adolesc Young Adul. 2024;13:577–582. 10.1089/jayao.2023.0138 PMC11296315 38451722

[24] Panagiotopoulou N Ghuman N Sandher R. Barriers and facilitators towards fertility preservation care for cancer patients: a meta-synthesis. Eur J Cancer Care (Engl). 2018;27:e12428. 10.1111/ecc.12428 26676921

[25] van den Berg M Baysal O Nelen WLDM. Professionals’ barriers in female oncofertility care and strategies for improvement. Hum Reprod. 2019;34:1074–1082. 31111876 10.1093/humrep/dez062

[26] Salsman JM Yanez B Snyder MA. Attitudes and practices about fertility preservation discussions among young adults with cancer treated at a comprehensive cancer center: patient and oncologist perspectives. Support Care Cancer. 2021;29:5945–5955. 33763727 10.1007/s00520-021-06158-0 PMC9023075

[27] Bastings L Baysal O Beerendonk CCM. Deciding about fertility preservation after specialist counselling. Hum Reprod. 2014;29:1721–1729. 24916435 10.1093/humrep/deu136

[28] Benedict C Thom B Kelvin JF. Young adult female cancer survivors’ decision regret about fertility preservation. J Adolesc Young Adul. 2015;4:213–218. 10.1089/jayao.2015.0002 PMC4684663 26697271

[29] Letourneau JM Ebbel EE Katz PP. Pretreatment fertility counseling and fertility preservation improve quality of life in reproductive age women with cancer. Cancer. 2012;118:1710–1717. 21887678 10.1002/cncr.26459 PMC3235264

[30] Kuntz H Santucci J Butts S. Determinants of decision regret regarding fertility preservation in adolescent and young adult cancer survivors: a systematic review. J Adolesc Young Adul. 2024;13:726–737. 10.1089/jayao.2023.0191 38717190

[31] Canzona MR Victorson DE Murphy K. A conceptual model of fertility concerns among adolescents and young adults with cancer. Psychooncology. 2021;30:1383–1392. 33843104 10.1002/pon.5695 PMC8363581

[32] Tschudin S Bitzer J. Psychological aspects of fertility preservation in men and women affected by cancer and other life-threatening diseases. Hum Reprod Update. 2009;15:587–597. 19433413 10.1093/humupd/dmp015

[33] Armuand G Wettergren L Nilsson J. Threatened fertility: a longitudinal study exploring experiences of fertility and having children after cancer treatment. Eur J Cancer Care (Engl). 2018;27:e12798. 29193408 10.1111/ecc.12798

[34] Parton C Ussher JM Perz J. Hope, burden or risk: a discourse analytic study of the construction and experience of fertility preservation in the context of cancer. Psychol Health. 2019;34:456–477. 30688090 10.1080/08870446.2018.1543764

[35] Rodriguez-Wallberg KA Ahlgren J Smedby KE. Prevalence and predictors for fertility-related distress among 1010 young adults 1.5 years following cancer diagnosis – results from the population-based Fex-Can Cohort study. Acta Oncol. 2023;62:1599–1606. 37909865 10.1080/0284186X.2023.2272291

[36] Anderson RA Brewster DH Wood R. The impact of cancer on subsequent chance of pregnancy: a population-based analysis. Hum Reprod. 2018;33:1281–1290. 29912328 10.1093/humrep/dey216 PMC6012597

[37] Madanat LMS Malila N Dyba T. Probability of parenthood after early onset cancer: a population-based study. Int J Cancer. 2008;123:2891–2898. 18798259 10.1002/ijc.23842 PMC2730156

[38] Lehmann V Ferrante AC Winning AM. The perceived impact of infertility on romantic relationships and singlehood among adult survivors of childhood cancer. Psychooncology. 2019;28:622–628. 30664284 10.1002/pon.4999

[39] Lehmann V Gronqvist H Engvall G. Negative and positive consequences of adolescent cancer 10 years after diagnosis: an interview-based longitudinal study in Sweden. Psychooncology. 2014;23:1229–1235. 24737637 10.1002/pon.3549 PMC4282587

[40] Quinn GP Huang IC Murphy D. Missing content from health-related quality of life instruments: interviews with young adult survivors of childhood cancer. Qual Life Res. 2013;22:111–118. 22286223 10.1007/s11136-012-0120-z PMC4291119

[41] Hawkey AJ Ussher JM Perz J. The impact of cancer-related fertility concerns on current and future couple relationships: people with cancer and partner perspectives. Eur J Cancer Care (Engl). 2021;30:e13348. 33084134 10.1111/ecc.13348

[42] Gorman JR Su HI Pierce JP. A multidimensional scale to measure the reproductive concerns of young adult female cancer survivors. J Cancer Surviv. 2014;8:218–228. 24352870 10.1007/s11764-013-0333-3 PMC4016119

[43] Gorman JR Drizin JH Malcarne VL. Measuring the multidimensional reproductive concerns of young adult male cancer survivors. J Adolesc Young Adul. 2020;9:613–620. 10.1089/jayao.2019.0146 32298593

[44] Shankara-Narayana N DiPierro I Fennell C. Sperm cryopreservation to insure fertility for men having gonadotoxic treatment: single centre experience over 4 decades. Clin Endocrinol (Oxf). 2018;89:59.

[45] Rodriguez-Wallberg KA Marklund A Lundberg F. A prospective study of women and girls undergoing fertility preservation due to oncologic and non-oncologic indications in Sweden-Trends in patients’ choices and benefit of the chosen methods after long-term follow up. Acta Obstet Gynecol Scand. 2019;98:604–615. 30723910 10.1111/aogs.13559

[46] Ferrari S Paffoni A Reschini M. Variables affecting long-term usage rate of sperm samples cryopreserved for fertility preservation in cancer patients. Andrology. 2021;9:204–211. 32814364 10.1111/andr.12894

[47] Fernández-González MJ Radauer-Plank AC Stelzer C. Sperm and testicular tissue cryopreservation and assisted reproductive technology outcomes in male cancer patients: a 15-year experience. J Cancer Res Clin Oncol. 2023;149:5321–5330; 2022. 36418559 10.1007/s00432-022-04488-y PMC10349735

[48] García A Herrero MB Holzer H. Assisted reproductive outcomes of male cancer survivors. J Cancer Surviv. 2015;9:208–214. 25272983 10.1007/s11764-014-0398-7

[49] Hammarberg K Kirkman M Stern C. Survey of reproductive experiences and outcomes of cancer survivors who stored reproductive material before treatment. Hum Reprod. 2017;32:2423–2430. 29045667 10.1093/humrep/dex314

[50] Ter Welle-Butalid ME Derhaag JG van Bree BE. Outcomes of female fertility preservation with cryopreservation of oocytes or embryos in the Netherlands: a population-based study. Hum Reprod. 2024;39:2693–2701. 39479806 10.1093/humrep/deae243 PMC11630040

[51] Ferrari S Paffoni A Filippi F. Sperm cryopreservation and reproductive outcome in male cancer patients: a systematic review. Reprod Biomed Online. 2016;33:29–38. 27156003 10.1016/j.rbmo.2016.04.002

[52] Lehmann V Both S Elzevier HW. “I wanna know what to expect”-Care needs regarding sexual and reproductive health after cancer in adolescence and young adulthood (AYA) and recommendations for providers. Eur J Oncol Nurs. 2025;74:102791. 39864240 10.1016/j.ejon.2025.102791

[53] Tong A Sainsbury P Craig J. Consolidated criteria for reporting qualitative research (COREQ): a 32-item checklist for interviews and focus groups. Int J Qual Health C. 2007;19:349–357. 10.1093/intqhc/mzm042 17872937

[54] Brooks J McCluskey S Turley E. The utility of template analysis in qualitative psychology research. Qual Res Psychol. 2015;12:202–222. 27499705 10.1080/14780887.2014.955224 PMC4960514

[55] Lindheim T. Participant validation: a strategy to strengthen the trustworthiness of your study and address ethical concerns. In: Espedal G, Jelstad Løvaas B, Sirris S, Wæraas A, eds. Researching Values: Methodological Approaches for Understanding Values Work in Organisations and Leadership. Chapter 13. Cham: Springer International Publishing; 2022:225–239.

[56] Francis JJ Johnston M Robertson C. What is an adequate sample size? Operationalising data saturation for theory-based interview studies. Psychol Health. 2010;25:1229–1245. 20204937 10.1080/08870440903194015

[57] Hillen MA Gutheil CM Strout TD. Tolerance of uncertainty: CONCEPTUAL analysis, integrative model, and implications for healthcare. Soc Sci Med. 2017;180:62–75. 28324792 10.1016/j.socscimed.2017.03.024

[58] Creux H Monnier P Son WY. Thirteen years’ experience in fertility preservation for cancer patients after in vitro fertilization and in vitro maturation treatments. J Assist Reprod Genet. 2018;35:583–592. 29502188 10.1007/s10815-018-1138-0 PMC5949117

[59] Daniluk JC Tench E. Long-term adjustment of infertile couples following unsuccessful medical intervention. J Couns Dev. 2007;85:89–100.

[60] Leiblum SR Aviv A Hamer R. Life after infertility treatment: a long-term investigation of marital and sexual function. Hum Reprod. 1998;13:3569–3574. 9886552 10.1093/humrep/13.12.3569

[61] Lehmann V Nahata L Ferrante AC. Fertility-related perceptions and impact on romantic relationships among adult survivors of childhood cancer. J Adolesc Young Adul. 2018;7:409–414. 10.1089/jayao.2017.0121 PMC6083209 29466084

[62] Morgan TL Young BP Lipak KG. “We can always adopt”: perspectives of adolescent and young adult males with cancer and their family on alternatives to biological parenthood. J Adolesc Young Adul. 2020;9:572–578. 10.1089/jayao.2020.0002 PMC7864105 32320315

[63] Canada AL Schover LR. The psychosocial impact of interrupted childbearing in long-term female cancer survivors. Psychooncology. 2012;21:134–143. 22271533 10.1002/pon.1875 PMC3123665

[64] Green D Galvin H Horne B. The psycho-social impact of infertility on young male cancer survivors: a qualitative investigation. Psychooncology. 2003;12:141–152. 12619146 10.1002/pon.622

[65] Crawshaw MA Glaser AW Hale JP. Male and female experiences of having fertility matters raised alongside a cancer diagnosis during the teenage and young adult years. Eur J Cancer Care (Engl). 2009;18:381–390. 19594609 10.1111/j.1365-2354.2008.01003.x

[66] Niemasik EE Letourneau J Dohan D. Patient perceptions of reproductive health counseling at the time of cancer diagnosis: a qualitative study of female California cancer survivors. J Cancer Surviv. 2012;6:324–332. 22752834 10.1007/s11764-012-0227-9

[67] Canzona MR Murphy K Victorson D. Fertility Preservation decisional turning points for adolescents and young adults with cancer: exploring alignment and divergence by race and ethnicity. JCO Oncol Pract. 2023;19:509–515. 37058685 10.1200/OP.22.00613 PMC10337714

[68] Vogt KS Hughes J Wilkinson A. Preserving fertility in women with cancer (PreFer): decision-making and patient-reported outcomes in women offered egg and embryo freezing prior to cancer treatment. Psychooncology. 2018;27:2725–2732. 30144212 10.1002/pon.4866

[69] Jardim FA Lopes-Junior LC Nascimento LC. Fertility-related concerns and uncertainties in adolescent and young adult childhood cancer survivors. J Adolesc Young Adul. 2021;10:85–91. 10.1089/jayao.2020.0058 32945713

[70] Ussher JM Perz J. Infertility-related distress following cancer for women and men: a mixed method study. Psychooncology. 2019;28:607–614. 30659694 10.1002/pon.4990

[71] Wettergren L Ljungman L Micaux Obol C. Sexual dysfunction and fertility-related distress in young adults with cancer over 5 years following diagnosis: study protocol of the Fex-Can Cohort study. BMC Cancer. 2020;20:722. 32758179 10.1186/s12885-020-07175-8 PMC7409491

[72] Dancet EAF Van Empel IWH Rober P. Patient-centred infertility care: a qualitative study to listen to the patient’s voice. Hum Reprod. 2011;26:827–833. 21317152 10.1093/humrep/der022

[73] Dong Y Zhang C Fang Y. Exploring strategies to alleviate reproductive concerns in cancer survivors: a comprehensive scoping review of international research. J Adolesc Young Adul. 2024;14:1–17. 10.1089/jayao.2023.0178 39231305

[74] Birt L Scott S Cavers D. Member checking: a tool to enhance trustworthiness or merely a nod to validation? Qual Health Res. 2016;26:1802–1811. 27340178 10.1177/1049732316654870

