# Supplementary material for: Are we Only Doing Good? Long-term Psychosocial Effects of Fertility Preservation (or Lack Thereof) on Survivors of Cancer During Adolescence and Young Adulthood
Source: Cancer J. 2025 Aug 11;31(4):e0774. doi: 10.1097/PPO.0000000000000774 (PMC12327501; doi:10.1097/PPO.0000000000000774)
Supplement: Supplementary file 1 [file ppo-31-e0774-s001.pdf]

Appendix to the manuscript:

Are we only doing good ? –
Long-term psychosocial effects of fertility preservation (or lack thereof) on survivors of AYA cancer

Vicky Lehmann, Niels van Poecke, Leah Waterman, Christianne Lok, Catharina Beerendonk, Ellen Smets

Data are part of the FROSA-study:
FROSA = <u>F</u>ertility, <u>RO</u>mance, and <u>S</u>ex in Young <u>A</u>dulthood

<u>Overview</u>

Appendix 1: English translations of example interview questions/ generic overview of topic list
Appendix 2: Schematic overview of identified themes and topics and their potential to change over time
Appendix 3: Additional example quotes

Appendix 1: English translations of example interview questions/ generic overview of topic list

If participants had **<u>completed</u>** fertility preservation

1. Impact of fertility preservation[1]

How has freezing your [sperm/eggs/embryo's/tissue][2] affected your life?
What do you know about your fertility now?
If not having children: Did or do you want to have children?
Did the fact that you completed fertility preservation - thus that you froze [sperm/eggs/embryo's/tissue][2] - ever influence your considerations whether you wanted to have children or not?
How?

2. Impact relationships & singlehood/ dating

<u>Partnered participants</u>	*<u>Unpartnered/ single participants:</u>*
How are your current or previous relationship(s) affected by the fact that you froze some of your material, that is [sperm/eggs][2]?<br>- and/or affected by possible infertility?<br><br>How did you discuss a (possible) **desire** to have children and that there is something **frozen** with your [current/previous] partner(s)?	How is your dating life (or previous relationships) affected by the fact that you froze some your material, that is [sperm/eggs][2]?<br>- and/or affected by possible infertility?<br><br>How did you discuss a (possible) **desire** to have children and that there is something **frozen** with your previous partner(s) in the past?

If <u>not</u> interested in having children:
How did the fact that you (probably) do not want to have children ever affected your [current/ previous] **relationships**?

---

[1] note that terms like fertility preservation, frozen material etc. have been explained to participants at the beginning of the interview and through written materials beforehand
[2] Adjusted to each participants' situation

If participants had **not completed** fertility preservation

1. **Impact of no fertility preservation**[1]

What does it mean for your life - at the moment - that you did *not* freeze any material?

What do you know about your fertility now?

If not having children: Did or do you want to have children?

Did or does the fact that you did *not* complete fertility preservation ever influence your considerations whether you wanted to have children or not?

How?

2. **Impact on relationships & singlehood/ dating**

Partnered participants	*Unpartnered/ single participants:*
How are your current or previous relationship(s) affected by the fact that you did not freeze any material, that is [sperm/eggs][2]?<br>- and/or affected by possible infertility?<br><br>How did you discuss a (possible) **desire** to have children and **fertility** with your [current/previous] partner(s)?	How is your dating life (or previous relationships) affected by the fact that you did not freeze any material, that is [sperm/eggs][2]?<br>- and/or affected by possible infertility?<br><br>How did you discuss a (possible) **desire** to have children and **fertility** with your previous partner(s) in the past?

If not interested in having children:

How did the fact that you (probably) do not want to have children ever affected your [current/ previous] **relationships**?

Appendix 2: Schematic overview of identified themes and topics and their possible change over time

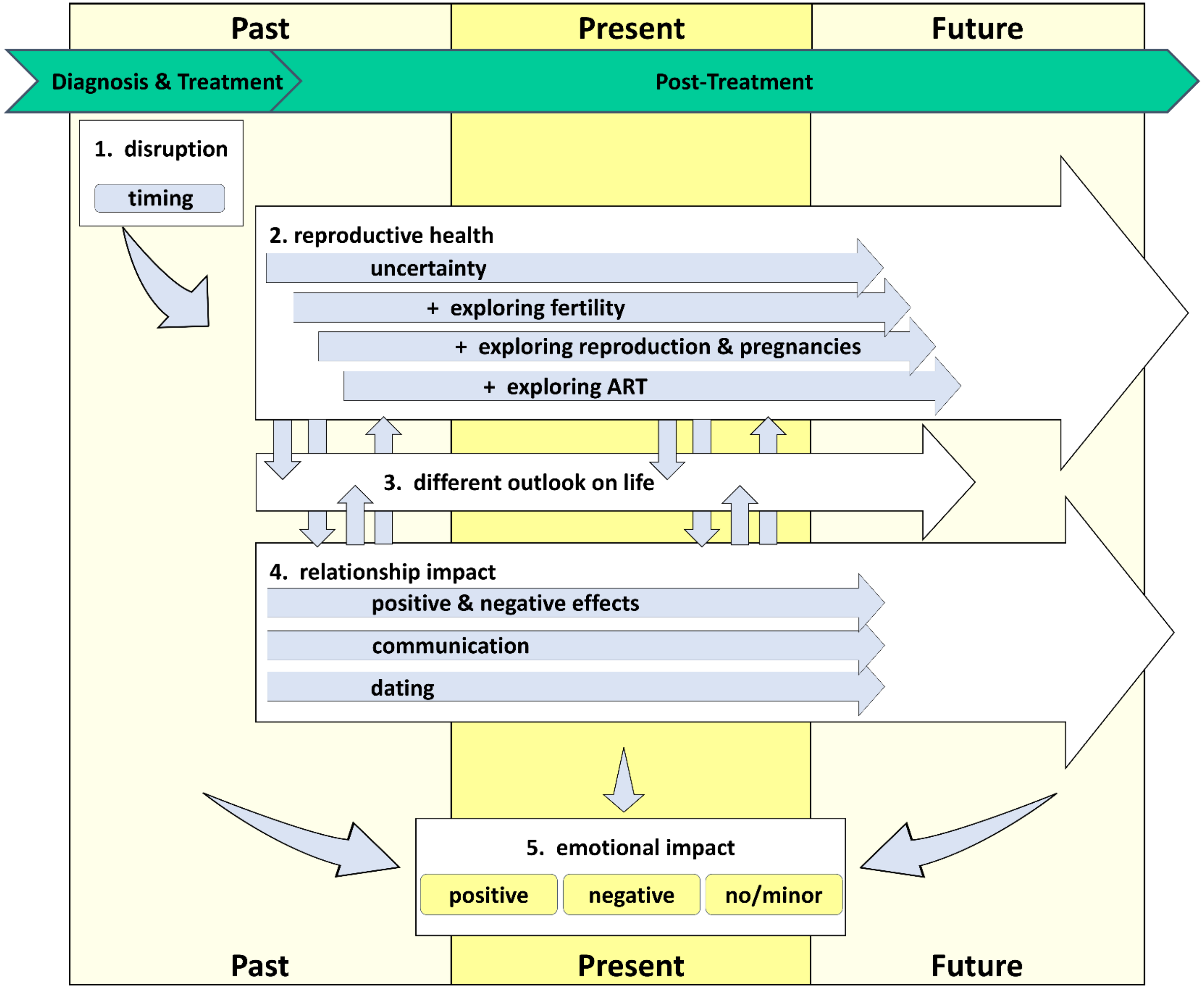

Appendix 3: Additional example quotes

1. Disruption of family planning

"[Me and my partner] had already agreed upon our desire to have children. So it was only put on hold and that was very difficult for me. I almost found that more difficult, because you're kind of putting off your future."

2. Reproductive Health following treatment

2.1 uncertainty

"I didn't feel like the decisions [for no fertility preservation] was that hard at the time. But in the years following, you have that uncertainty: am I still fertile or not?" [4024]

"[frozen material] is kind of an insurance for me; cause I still have that strong desire to have children. By now, I'm menopausal since almost 8 years […] thus, it's unclear if I am still fertile. I'll find out in a about 2 years." [4003]

"You'll only find out after the [hormonal]therapy if your fertility will recover. It's almost a pervert incentive to stop with hormone therapy." [4010]

→ **2.2 exploring fertility** (testing, contemplate alternatives)

"At the time, I figured I'm still young, but that I should check just to make sure. I think I was around 24-25 […] and I was infertile, and we immediately discussed that we shouldn't wait for too long putting our plans to have children into action [through IVF]" [4015]

"I was done with treatment for a couple of months and got some menopausal symptoms; hot flushes and stuff. So I said I want to have that tested […] and it became clear that I was going through menopause. My female hormones were so low, they couldn't be measured. My oncologist said that it might recover within 3 years. -but due to my age, I might be a borderline case. Above 35, it typically doesn't recover, under age 35 there is a big chance that it recovers. Thus, I assume that my chance of recovery is small, but he thinks it can recover within 3 years." [4027]

"You're kind of getting only 10% of the information [from providers]; like "now we've given you a little hope". But the feasibility [of alternatives], that is actually much worse. If you look into it […] you actually find out that very little is possible. And that is yet another blow." [4006]

→ **2.3 exploring reproduction & pregnancies**

"We wonder, if I can handle [another child] -together with my symptoms of fatigue. And of course, [I contemplate] if I dare to go through the whole [ART] process again […]. Aren't you increasing your risk [for cancer] again? Especially if you're temporarily stopping with hormone therapy again […], and how far would you go? […] if it doesn't work, would you leave it at [IUI] or would you go from IUI to IVF? - because that are also quite heavy treatments." [4011]

"We're trying to get pregnant. And it's not working [naturally]. So my oncologist decided that I can get help [through ART]. […]. He said quite bluntly that he wants me to give birth and go back on hormone therapy. This is quite intense, especially since it didn't work yet to get pregnant." [4033]

→ **2.4 exploring ART**

"I simply have a desire to have children, but nothing has to happen from my side anymore [to sire a pregnancy because sperm is frozen]. And I put some kind of pressure on a partner later. On a woman. […]. I find this difficult. I don't want to burden anyone [with IVF]" [4009]

"[with ex-partner] been to the hospital for 6 years to try and have a baby [through ICSI], but it never worked." [4004]

3. Different future and outlook on life

"That you lose your hair […] that doesn't matter […] I had this idea about how [my future] would look like …and now that's off the table. That was real tough. I was very sad about it. About cancer -not so much. There's treatment for it and it went well … but then suddenly, your whole vision of the future looks differently." [4001]

"I talked to my husband about it yesterday: if my period comes back, would you want to try and get pregnant again over sometime? And we both think this is still a difficult question, because we have gone through so much […] He says he's OK with how things are; with 1 child. On the other hand, I think that a joyful postnatal period has been taken away from me. My daughter's first year in life was dominated by [cancer]. And I would actually love to have a do-over." [4027]

4. Relationship Impact

4.1 positive and negative effects

"Overall, I think it brought us closer together. I think relationships can turn one way or the other. That you either lose touch and don't talk or that you just expose your vulnerabilities and that that actually connects you to each other […] Regarding our reproductive goals, I also think that we moved forward with that together. It wasn't just about my wishes or solely about his wishes, but we really weighed it together. It is not only my choice. He is just as much a part of it." [4005]

"At the time of diagnosis, there wasn't really a pressing desire to have children, but that [my partner] would want it the future. -But if it doesn't work, well then it doesn't. He would be fine with that too. Yet, he changed his mind over time […] That is also one of the reasons why he ended the relationship. […] Initially I was happy about his reaction. He was also OK with [IVF] and maybe even adoption. But he backtracked." [4031]

4.2 partner communication

"We had various conversation about [infertility] and that was pretty tough, because you might not be ready for it, but you have to address it. And he actually indicated that his desire to have children was not bigger than the love he has for me. That was very beautiful. But I also have to honestly admit that -in the back of mind- I'm thinking yes that's the way it is for now, but you can never look into the future." [4013]

4.3 dating

"I thought, at least I'm fertile. It's silly, but I felt inferior, like a 'problematic case'. Who wants to be in a relationship with someone who had cancer, which might even come back? So then -at least- I am still able to have children. It's a little sad, but that was playing in the back of my mind." [4010]

"If I want to get pregnant, it will be quite complex. It's not like you decide this together and stop using protection and just try." [4048]

"Yeah, when do you tell? […] that question will pop up eventually. So I thought before you are deep into the dating process and kinda have a real relationship […], I'll have to be upfront like 'sorry, but if you want to have children, you better be […] not with me. […] you really have to be open and honest." [4035]

5. Emotional impact

5.1 Positive feelings

"I'm really happy with [preservation] because it gives me space and reduces pressure when it comes to a possible desire to have children […] actually, the fact that there is something in the freezer takes away the consequences [of fertility problems]. It's like ok, de natural way could be very difficult. It's not impossible, but difficult, and then we always still have the possibility to use the good [frozen] sperm." [4018]

"The fact that I have something in the freezer, was always plan B. Like okay,… the treatment is done; if I am fertile or not, I don't know. and we'll see about that, once the desire to have children gets more concrete. […] and we actually found out last year that I indeed became infertile due to the treatment. And that was, […] quite a blow that we actually need [this plan B]." [4025]

5.2 Negative feelings

"Since I had [cancer], I started looking at [the desire to have children] in a total different way. [Fertility] is not a given. So I'm happy for my friends, maybe even more so than before. Because now you know how it feels when it's not possible anymore. But for me, it is -nevertheless- confronting and difficult." [4013]

5.3 Minimal/absent emotional impact

"I honestly don't have the feeling that [no preservation] had a lot of effect. We were only busy surviving. That was most important; and we were extra thankful about the son that we already had. But we are sometimes thinking like 'hey, how would it have been to have another child?' but -luckily- I don't have the feeling that it changes anything." [4036]

"Look, I'm still young. I don't have a partner now, so I'm not thinking about [frozen material] and having children" [4010]
